# Development and psychometric evaluation of the Spanish Cooking Self-Efficacy Questionnaire (SCSEQ) for Spanish University Students

**DOI:** 10.1371/journal.pone.0352758

**Published:** 2026-07-08

**Authors:** Patricia Jurado-Gonzalez, F. Xavier Medina, Alba Martínez-Garcia, Almudena López-Cediel Verdú, Sergi Fàbregues, Anna Bach-Faig

**Affiliations:** 1 NUTRALiSS, Faculty of Health Sciences, Universitat Oberta de Catalunya (Open University of Catalonia, UOC), Barcelona, Spain; 2 Epi4Health Research Group, Faculty of Health Sciences, Universitat Oberta de Catalunya (Open University of Catalonia, UOC), Barcelona, Spain; 3 UNESCO Chair on Food, Culture and Development, Universitat Oberta de Catalunya (Open University of Catalonia, UOC), Barcelona, Spain; 4 Department of Community Nursing, Preventive Medicine, Public Health and History of Science, University of Alicante, Alicante, Spain; 5 Talent Data Scientist, Data Scientist and Psychometric Specialist, Madrid, Spain; 6 Department of Psychology and Education, Universitat Oberta de Catalunya (Open University of Catalonia, UOC), Barcelona, Spain; Lusofona University of Humanities and Technologies: Universidade Lusofona de Humanidades e Tecnologias, PORTUGAL

## Abstract

The transition to university is a critical life stage characterized by increased autonomy, identity exploration, and new social and environmental influences. During this period, university students often exhibit low adherence to dietary guidelines. Among the determinants influencing healthy eating, cooking self-efficacy, the central construct of Social Cognitive Theory (SCT), is consistently associated with improved diet quality and is a frequent target of health interventions. However, no validated instrument exists to assess this construct among university students in Spain. Therefore, the goal was to develop and provide preliminary evidence of the Spanish Cooking Self-Efficacy Questionnaire (SCSEQ), a concise SCT-based instrument tailored to Mediterranean university settings. A 32-item questionnaire was developed through a review of existing instruments assessing cooking self-efficacy. Face validity was evaluated with Spanish food and nutrition experts (n = 12) to assess the clarity and pertinence of the initial items. The revised Spanish Cooking Self-Efficacy Questionnaire (SCSEQ) was then pilot-tested with Spanish university students (n = 73) from four Catalan universities. Exploratory factor analysis (EFA) was conducted to identify the underlying factor structure and detect problematic items. Internal consistency reliability was assessed using McDonald’s ω, and test–retest reliability over a two-week interval was evaluated using Pearson correlations. Face validity indicated overall clarity and adequacy. Four items were excluded and recombined, two items were added, and nine items were rewritten based on experts’ feedback. After pilot testing, the questionnaire overall demonstrated high internal consistency (ω = 0.9). Items were reviewed based on factor loadings, item redundancy, theoretical relevance, and their contribution to scale-level internal consistency. EFA suggested a two-factor structure with good internal consistency (ω = 0.88 and ω = 0.82) and test–retest reliability (ICC = 0.91, 95% CI [0.80, 0.96]). Three items with weak loadings were excluded. The final version consisted of 25 items and 2 subscales. The SCSEQ showed favorable preliminary psychometric properties.

## Introduction

The transition to university is a critical period for shaping lifelong dietary habits, as young adults navigate substantial personal, social and environmental changes. For some students, this period entails living independently for the first time, while for others it involves a more gradual increase in autonomy over food choices, schedules, social eating practices, and health-related decisions, even when they continue to live in the family home [1]. However, this transition is often accompanied by a decline in diet quality. University students consistently report limited adherence to national dietary recommendations [2–4]. Their diets are typically low in vegetables, fruits, legumes, whole grains, and fish, while being high in ultra-processed foods, sugary drinks, and alcohol consumption [5,6]. Dietary patterns such as skipping meals, frequent snacking, and irregular eating schedules are also common [7]. Since dietary habits formed during this life stage often persist into adulthood [8], this nutritional decline has become a growing public health concern.

Nevertheless, understanding how to improve dietary behaviours among university students requires a systems perspective that considers the multiple, interacting determinants shaping their food-related decisions. These determinants operate across individual, interpersonal, environmental, and sociocultural levels, including perceived lack of time, academic stress, low motivation for healthy eating, limited food literacy and cooking self-efficacy, peer and family influences, affordability constraints, access to adequate cooking facilities, availability of healthy and convenient food options on or around campus, and broader norms that normalize convenience foods, eating out, and irregular eating patterns.

Among these determinants, cooking self-efficacy, defined as the belief in one’s ability to plan and prepare meals using a variety of ingredients and techniques [9–14], has emerged as a particularly modifiable individual-level factor with a strong influence on dietary behaviour [15]. It is especially relevant in university populations because it may shape how students respond to common barriers to healthy eating, including perceived lack of time, stress, low motivation, limited budgets, shared cooking facilities, and unfamiliar ingredients. Unlike broader structural determinants, such as food prices or university food environments, cooking self-efficacy can be directly strengthened through culinary education [16]. Moreover, evidence consistently shows that higher cooking self-efficacy is associated with more frequent home meal preparation, improved dietary variety, greater intake of fruits and vegetables, and an enhanced ability to overcome external barriers to healthy eating [17–19]. Consequently, there is a growing consensus in the literature supporting the implementation of culinary interventions that go beyond teaching skills to enhance cooking self-efficacy among university students as a strategy to improve nutrition-related outcomes and overall wellbeing [4,20,21].

At the core of this approach lies Social Cognitive Theory (SCT), one of the most widely used behavioural frameworks in health education. Developed by Bandura (1986), SCT conceptualizes behaviour as the result of dynamic interactions between personal, environmental, and behavioural factors. Relevant to the theory is the construct of self-efficacy, which refers to an individual’s belief in their capacity to execute actions necessary to achieve desired outcomes. This belief influences motivation, goal-setting, perseverance, and behavioral regulation [22,23]. In the context of cooking, it involves having the confidence to cook even with limited time, shared kitchen space, or unfamiliar ingredients [16,19,24]. Moreover, SCT provides behavioural strategies to enhance self-efficacy during interventions, emphasizing that confidence can be improved through mastery experiences, social modelling, and verbal encouragement within supportive environments, making it a practical framework for culinary education [9,17].

Despite the growing conceptual and empirical evidence supporting the role of cooking self-efficacy in improving diet quality and overall health indicators, there is no validated instrument designed specifically to measure cooking self-efficacy among Spanish university students in a concise and targeted format suitable for use in digital culinary and nutrition interventions. Most of the existing instruments to measure culinary outcomes were developed in sociocultural contexts where cooking practices, ingredient familiarity, and food language differ significantly from those in Mediterranean cultures [4,9]. This limits their relevance and sensitivity in Spanish settings. Moreover, many existing instruments are lengthy, overly broad, incorporate overlapping domains such as dietary intake, or are grounded in alternative behavioral frameworks such as Self-Determination Theory (SDT) [25,26]. Moreover, most were developed for in-person instruction and lack adaptability for digital or hybrid interventions now common in university settings [4,9,25–33].

In light of these gaps and considering the sociocultural and educational context of Spanish university students, this study aimed to develop the Spanish Cooking Self-Efficacy Questionnaire (SCSEQ) and conduct a preliminary psychometric evaluation among Spanish university students, including face validity, internal consistency, exploratory structural validity, and test–retest reliability. The objective was to create a concise, theory-informed instrument tailored to the Mediterranean context to accurately assess cooking self-efficacy and support the evaluation of dietary interventions promoting health among Spanish university students.

## Materials and methods

**Fig 1:**
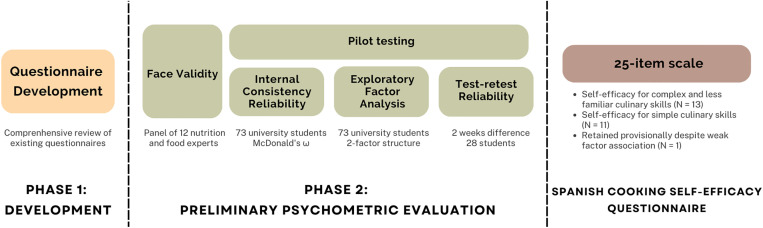
Development and evaluation phases and subphases of the Spanish Cooking Self-Efficacy Questionnaire.

The development and preliminary psychometric evaluation of the SCSEQ were conducted in two phases (Fig 1): questionnaire development and psychometric evaluation. The evaluation was further divided into two subphases: face validity testing with nutrition and food experts, and pilot testing with university students. During the pilot testing phase, several psychometric analyses were conducted. These included assessing internal consistency (Cronbach’s alpha), conducting an exploratory factor analysis (EFA) to examine the latent structure, and calculating the internal consistency of the identified factors. Additionally, test–retest reliability was evaluated to assess the questionnaire’s temporal stability.

### Phase 1: Questionnaire development

The initial version of the SCSEQ was developed through a comprehensive review of validated instruments aimed at assessing cooking self-efficacy among university students and other sociodemographic groups. While the selected items were not exclusively drawn from instruments specific to university students, the item selection process was intentionally guided by the skills and constraints most relevant to this group. The goal was to capture the specific skills university students need to feel confident in organizing, planning, and preparing meals on a regular basis, particularly under real-life constraints [12,14,15]. These skills include applying common cooking techniques, cooking with key food groups (e.g., vegetables, legumes, cereals, fish, fruits), and feeling capable of organizing and preparing meals when time, space, or resources are limited. This approach ensured that the SCSEQ targeted concrete, modifiable behaviours, increasing its theoretical rigor and practical relevance for future culinary interventions.

The initial review covered a broad range of instruments, but the final pool of items was primarily drawn from three core sources. Two of these were the instruments developed by Lavelle, Spence, et al., (2016) [34], and later refined by Costa et al., (2023) [29], which provided the structural foundation for the SCSEQ. These instruments were not originally designed for university students, but they offered clear, practical items for assessing perceived cooking and food-preparation skills, particularly those related to culinary techniques, planning, and organizational tasks. From these sources, items were adapted for relevance to university students. Items were excluded according to three criteria. First, conceptually overlapping items, such as “planning meals ahead” and “preparing food in advance”, were merged to enhance clarity and reduce redundancy. Second, items that represented culinary competencies that were too complex or not well adapted to the Spanish university context, based on previous qualitative research with students [12], were also excluded. For example, the item “*make sauces and gravy from scratch*” was excluded due to its limited relevance and perceived difficulty among students. Third, items that did not align with the core construct of cooking self-efficacy, such as those related to nutrition knowledge or food label reading, were excluded to maintain theoretical focus.

Given that the original instruments targeted broader sociodemographic groups, this selective adaptation was essential to ensure contextual relevance. All final items were translated into Spanish and adapted to Mediterranean food practices by the lead researcher (PJG), a native Spanish speaker with culinary expertise in Mediterranean cuisine.

The third key source that was used to identify the initial pool of items was the latest version of the Cooking With a Chef (CWC) questionnaire [9”], an instrument explicitly grounded in SCT. Items were selected from three subscales included in this instrument: Cooking Self-efficacy (SE) Scale, Self-Efficacy for Using Basic Cooking Techniques (SECT) Scale, and Self-Efficacy for Using Fruits, Vegetables, and Seasonings (SEFVS) Scale. Other scales in the instrument, such as the Availability and Accessibility of Fruits and Vegetables (AAFV) Index, were excluded to preserve theoretical alignment, as they were beyond the scope of the SCSEQ. The selected items were translated and refined to enhance clarity and relevance for Spanish students with limited culinary experience. For example, general terms such as “stewing” or “grilling” were rephrased to provide more detailed and accessible descriptions within the Mediterranean context. Specifically, “stewing” was rewritten as “*To stew (to cook for long periods of time, at least one hour, in a liquid or sauce, as in beef stew)*”. Moreover, the SEFVS subscale was substantially expanded. While the SEFVS focused only on fruits, vegetables, and spices, additional food groups commonly used in the Mediterranean context, such as legumes, cereals, and fish, were incorporated. All adaptations were performed by the lead researcher to ensure conceptual consistency with the SCT and contextual appropriateness for Spanish university students.

The resulting initial version of the SCSEQ comprised 32 items organized in three subscales (see S1 File). The first subscale labelled “General,” comprised 7 items assessing overall cooking self-efficacy and confidence in managing common cooking situations. The second subscale included 19 items and was divided into two domains: “Culinary Techniques,” which assessed self-efficacy in performing specific cooking methods (e.g., boiling, steaming, frying), and “Food Groups,” which evaluated confidence in preparing key food categories relevant to the Mediterranean diet (e.g., vegetables, legumes, fish). The third subscale consisted of 6 items focusing on two related areas: “Culinary Resources,” which captured the ability to creatively use available ingredients and leftovers, and “Culinary Organization,” which assessed planning and time management skills in relation to meal preparation. A 5-point Likert scale was chosen instead of a 7-point format based on prior research indicating that 7-point response options may lead to threshold violations or increased response burden, particularly in younger populations with limited time or experience in survey research [31].

### Phase 2: Psychometric evaluation

The full psychometric evaluation process of the SCSEQ was done during May and June 2024.

#### Face validity.

This phase aimed to confirm that the initial item pool accurately measured the intended construct and that all questions were clear, pertinent, and adequate [30]. Face validity was assessed in May 2024 by a panel of 12 independent experts in food and nutrition with professional or academic experience in the Spanish and/or Mediterranean context. The experts were selected based on their academic and/or professional expertise in nutrition science, public health, or culinary instruction, as well as familiarity with the dietary habits and food-related challenges faced by university students in Spain. Each expert received a copy of the first version of the questionnaire via email and was asked to evaluate the clarity and pertinence of each item on a scale from 1 to 3 (1 = not clear, 3 = clear). Additionally, they were asked to provide suggestions and additional comments for enhancing item clarity, considering the typically low culinary knowledge among university students and the relevance of the items to Mediterranean practices. Items rated as unclear or irrelevant (scores of 1 or 2) were either revised or removed based on both the experts’ quantitative ratings and qualitative comments. This process resulted in the development of the second version of the SCSEQ (see S2 File).

### Pilot testing and data collection

#### Participants and procedures.

In May 2024, a total of 73 students were recruited through institutional email lists from four Catalan universities: *Universitat de Barcelona (UB), Universitat de Girona (UDG), Universitat Autònoma de Barcelona (UAB), and Universitat Pompeu Fabra (UPF)*. The recruitment process was stratified by degree program to ensure diversity. Eligibility criteria included: (1) being between the ages of 18 and 22; (2) living off campus; and (3) being fluent in Spanish. To reduce potential bias associated with higher cooking self-efficacy, participants enrolled in a nutrition or culinary degree program were excluded. Students participating in university meal plans were also excluded. No compensation was offered for participation.

#### Test-retest reliability.

Test-retest reliability was evaluated in a subsample of participants (n = 28) over a 14-day interval, a commonly used timeframe that balances the risk of memory effects with the assumption of construct stability [30]. This approach aimed to assess the temporal stability of the SCSEQ under consistent conditions and in the absence of external interventions, as recommended for constructs expected to remain stable in the short term, such as cooking self-efficacy [35]. All 73 students who completed the initial questionnaire (Time 1) were invited via email to participate in a second administration (Time 2) two weeks later. Participation in the retest was voluntary. Intraclass correlation coefficients (ICC) with 95% confidence intervals were calculated as the primary indicator of test–retest reliability, as ICC assesses agreement between repeated measurements rather than only the strength of association. Pearson correlations were also reported to allow comparison with previous studies, but they were interpreted as supplementary indicators. Correlations above 0.80 were considered as evidence of excellent reliability, indicating high temporal consistency [35]. To assess whether voluntary dropout introduced systematic bias, Time 1 SCSEQ scores and demographic characteristics (age, gender, living arrangement) were compared between retest completers and non-completers using independent samples t-tests and chi-square tests, respectively. Given the relatively small retest subsample, all temporal stability estimates were interpreted cautiously.

### Statistical analysis

All statistical tests were two-sided, and differences at *p* < 0.05 were considered significant. All statistical analyses were performed using the statistical software package Jamovi (Version 2.5.4.0), a free, open-source statistical program.

#### Internal consistency reliability.

Internal consistency was evaluated using McDonald’s ω [36] and Cronbach’s *α* [37,38]*,* both of which were reported with 95% confidence intervals (CI). These indices were used to evaluate the degree to which items on the SCSEQ consistently measured the same underlying construct after the first administration of the questionnaire. McDonald’s ω was selected as the primary reliability index because it is derived from a factor analytic approach and is considered more robust than Cronbach’s α [38,39]. A threshold of ω ≥ 0.70 was used to indicate acceptable internal consistency [39]. Items were reviewed through an iterative process considering their contribution to overall scale reliability, corrected item–total correlations, conceptual redundancy, theoretical relevance to cooking self-efficacy, and clarity based on previous expert feedback. For instance, items with ω < 0.70 were removed unless they measured unique aspects of cooking self-efficacy (e.g., planning under time constraints) [40]. This iterative process was used to refine the questionnaire before the exploratory factor analysis. The resulting version of the SCSEQ was then submitted to EFA to examine its preliminary latent structure (see S3 File) [30].

#### Construct validity.

EFA was conducted to examine the latent factor structure underlying the SCSEQ, in alignment with the theoretical construct of cooking self-efficacy [35]. Given the developmental stage of the SCSEQ and the absence of a previously established factor structure for this instrument, EFA was considered an appropriate method for examining its underlying dimensionality. Data suitability for EFA was confirmed by a statistically significant Bartlett’s test of sphericity (χ² = 938, df = 378, p < 0.01), indicating the correlation matrix was appropriate for factor analysis [41,42]. The optimal number of factors was determined through Horn’s Parallel Analysis (PA), which compares the dataset’s eigenvalues with those from randomly generated correlation matrices of equal size [35]. Factors were retained when the observed eigenvalues exceeded the 95th percentile of the simulated distribution [43,44]. The simulation was conducted using a Monte Carlo approach to incorporate sampling variability and enhance the robustness of PA [43].

Factor extraction was carried out using the minimum residuals method. Oblimin rotation, an oblique technique suitable for correlated factors, was applied to reduce cross-loadings [26]. Item retention was guided by a combination of empirical and theoretical criteria, including factor loadings, cross-loadings, conceptual redundancy, clarity, and relevance to the construct of cooking self-efficacy. Factor loadings ≥0.50 were considered strong indicators of factor membership, while items with lower loadings were reviewed individually and retained only when they contributed meaningfully to content coverage and theoretical coherence. The EFA was repeated after removing the weak items to confirm the factor structure. McDonald’s ω was then calculated for each factor to assess the internal consistency reliability of the identified subscales [45].

### Ethical considerations

This study was conducted according to the guidelines laid down in the Declaration of Helsinki and all procedures involving research study participants were approved by the Institutional Review Board of the *UOC* (CE23-TE04). Each participant in the face validity process and in the survey received and signed a written informed consent form. Students were assured that their personal information would remain confidential.

## Results

The demographic characteristics of the participants involved in the face validity and pilot testing phases are summarized in Tables 1 and 2. In the pilot testing phase, a total of 73 students completed the questionnaire in its entirety and were included in all analyses. While 80 survey responses were initially recorded in the data collection platform, seven participants were excluded because they did not complete all items or did not meet the eligibility criteria, resulting in a final analytic sample of 73 participants. See S5 File for the full dataset.

**Table 1:** Demographics of expert participants in the face validity testing subphase.

Characteristic	Total number (%)
***Gender***	
Male	1 (8%)
Female	11 (92%)
***Age***	
23–30 years	3 (25%)
> 30 years	9 (75%)
***Background***	
Food scientists	2 (17%)
Chefs	2 (17%)
Nutrition experts	8 (67%)

**Table 2:** Demographic characteristics of student participants in the pilot testing subphase.

Characteristic	Total number (%)
***Gender***	
Male	21 (28%)
Female	48 (65%)
Prefer not to say	3 (4%)
***Age***	
18–19 years	28 (38%)
20–21 years	31 (42%)
≥ 22 years	15 (20%)
***Degree program***	
Health Sciences	34 (46%)
Psychology & Social Work	9 (12%)
Biology & Environmental Sciences	9 (12%)
Law, Education & Pedagogy	8 (11%)
Engineering & Computer Science	8 (11%)
Other	6 (8%)
***Living arrangement***	
With family	53 (72%)
With other students	17 (23%)
Alone	3 (4%)

### Face validity

After evaluating the responses provided by the expert panel, two items in the questionnaire were modified and merged to improve clarity and relevance. The item *“When I cook, I feel prepared to manage any unanticipated event”*, was considered potentially confusing for individuals with limited cooking experience. Therefore, it was merged with a similar item, *“When I cook, I feel that I can solve any issues that arise with little effort.”* The second modified item, which originally referred to *“rice”* as a distinct food group, was revised to *“cereals”* to more accurately reflect typical Mediterranean dietary classifications and to encompass both pasta and rice.

Additionally, based on the panel’s feedback (see S2 File), eight items were reworded to enhance clarity. Two new items were also introduced: one assessing confidence in preparing “*fruit”* as a food group and another evaluating the culinary technique of “*grilling in the pan”*. These expert-informed adjustments led to a revised pool of 32 items, which was used for subsequent pilot testing among Spanish university students.

### Internal consistency reliability

The initial version of the questionnaire showed high scale-level internal consistency, with a McDonald’s ω coefficient of 0.89. As part of the item refinement process, four items were identified as having weak empirical performance (e.g., Culinary Resources) [3], including low factor loadings (<0.30), limited contribution to scale-level reliability, and/or conceptual overlap with better-performing items. These items were reviewed considering their corrected item-total correlations, contribution to McDonald’s ω if deleted, conceptual redundancy, clarity, and relevance to the construct of cooking self-efficacy.

One additional item, assessing confidence in using the microwave for cooking, also showed weak empirical performance. However, because microwave use may represent an accessible and commonly used cooking method among university students [12]. This item was retained provisionally and reworded for greater clarity as follows: “*Cook with the microwave (steam cooking, Lékué, grill, etc.)*.” Following item removal and revision, the overall internal consistency of the refined questionnaire slightly improved from ω = 0.89 to ω = 0.90, indicating strong scale-level coherence among the retained items before EFA.

### Construct validity

**Fig 2:**
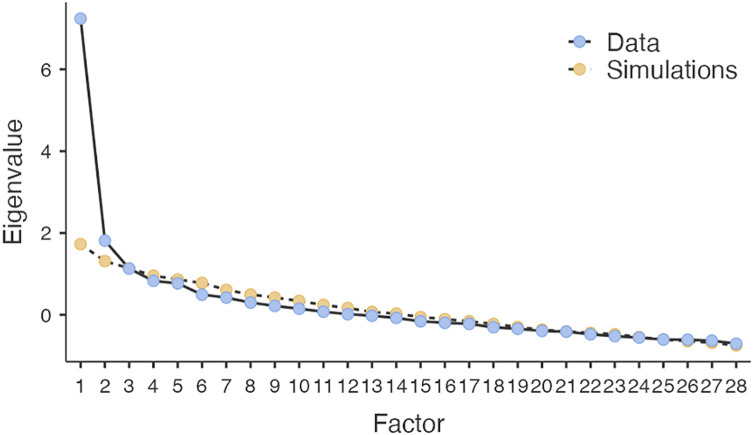
Parallel analysis scree plot comparing eigenvalues from actual data and simulated data (n = 73).

The EFA suggested a two-factor structure (Table 3), as supported by Horn’s parallel analysis and the scree plot (Fig 2). The KMO value was 0.74, indicating acceptable sampling adequacy for exploratory factor analysis, and Bartlett’s test of sphericity was statistically significant (χ² = 938, df = 378, p < 0.01), indicating that the correlation matrix was suitable for factor analysis. However, given the small sample size, the KMO value should be interpreted as indicating the minimum threshold for acceptable adequacy rather than strong evidence of factor stability.

**Table 3:** Self-Efficacy item groupings, factor loadings, and reliability coefficients for the final SCSEQ version (n = 73).

Coding	Items	Factors		
		1	2	Uniqueness (1 – communality)
General_2	I feel limited in the kitchen due to my lack of culinary knowledge	0.703		0.436
General_3	I feel capable of cooking with the ingredients I have at home	0.493		0.701
General_4	When I cook, I feel prepared to handle problems or incidents that arise, such as missing an ingredient from the recipe and having to improvise	0.419	0.437	0.494
General_6	I feel prepared to read or watch and try new recipes	0.322	0.354	0.698
Food groups_1	Vegetables	0.794		0.450
Food groups _3	Cereals (pasta, rice, quinoa, etc.)		0.484	0.706
Food groups _4	Tubers (potato, sweet potato or beet)	0.619		0.538
Food groups _5	Legumes (including soy and derivatives)	0.739		0.492
Food groups _6	Meat		0.586	0.687
Food groups _8	Fish		0.458	0.671
Food groups _9	Eggs		0.543	0.697
Culinary techniques_1	Stew (cook for long periods of time, at least an hour in a liquid or sauce). Example: beef in sauce	0.792		0.410
Culinary techniques _2	Boil (any type of food: rice, pasta, eggs, etc.)		0.480	0.728
Culinary techniques _3	Steam cooking (the food never touches the water, it is made with the steam itself)	0.594		0.570
Culinary techniques _4	Cook with the microwave (steam cooking, lekue, grill, etc.)			0.958
Culinary techniques _5	Roasting food in the oven (raw meat or fish, vegetables, etc.)		0.570	0.714
Culinary techniques _6	Fry in a pan or wok with oil (potatoes, vegetables, breaded meat, etc.)		0.575	0.661
Culinary techniques _7	Grill (cook on a very hot surface with little oil)		0.594	0.688
Culinary techniques _8	Pre-prepare raw vegetables (peel and cut an onion, a carrot, prepare a broccoli for cooking, etc.)	0.484		0.676
Culinary techniques _9	Pre-prepare meat (debone a chicken, make burgers, make meatballs, etc.)		0.472	0.771
Culinary techniques _10	Pre-prepare fish (remove the bones, clean it and prepare it for cooking)	0.396		0.731
Culinary techniques _11	Dress or season food (use herbs or spices to flavor dishes, prepare vinaigrettes and/or sauces)	0.590		0.456
Culinary resources _2	I feel capable of preparing healthy dishes with few ingredients	0.378		0.872
Planning and organizing _1	I feel capable of cooking one day for the entire week	0.502		0.785
Planning and organizing _2	I feel able to properly plan the food shopping list based on what I want to cook	0.438		0.756

Exploratory factor analysis of the final SCSEQ version after removing weak-loading and conceptually overlapping items. Items are grouped by their original thematic category. Factor loadings and item uniqueness values are reported. Factor 1 was interpreted as advanced cooking self-efficacy and Factor 2 as basic cooking self-efficacy.

Factor 1 appeared to reflect advanced cooking self-efficacy, encompassing confidence in executing more complex culinary tasks. This included techniques such as stewing, steaming, and preparing fish; managing less familiar or underused food groups among university students, such as legumes and fresh vegetables; and engaging in planning and resource management activities like scaling recipes or using leftovers. These items represent higher-order cooking competencies that require greater experience, planning, and adaptability. The factor showed strong internal consistency (ω = 0.88), supporting its reliability.

Factor 2 captured basic cooking self-efficacy, referring to confidence in performing foundational culinary tasks. This included simpler techniques, such as grilling or boiling and handling familiar staple foods such as cereals and eggs. These items reflect essential, routine skills typically used in day-to-day student cooking contexts. This factor also demonstrated good internal consistency (ω = 0.82).

These findings differed from the initial tripartite theoretical model, which organized items into general culinary competencies, specific culinary techniques, and organizational skills (S1 File). Instead, the exploratory two-factor structure suggested that students’ cooking self-efficacy may be characterized by perceived task complexity rather than by predefined content domains.

Several items showed weak loadings (< 0.3) or did not clearly associate with either factor: General [5], Culinary techniques [4], Culinary resources [1], and Culinary resources [4] (see S3 File for more context on those items). To maintain the final scale’s parsimony without compromising its content validity, three items were eliminated due to weak empirical performance (<0.3) and conceptual overlap with retained items [46–48]. The first item, General 5, which assessed whether *“In my kitchen, I have all the utensils I need to make any kind of recipe,”* was excluded because it primarily reflected a structural barrier to cooking rather than self-efficacy. The second item, Culinary resources 4, *“I feel able to use leftover food from other days to create a dish,”* was also removed due to conceptual redundancy with General 5, which already assessed students’ ability to cook with available ingredients. Similarly, Culinary resources 1, *“I feel capable of adapting recipes to the amount of food I want to prepare,”* was excluded because it overlapped with Planning and Organizing 1, which addressed comparable skills related to recipe scaling.

However, despite its weak loading, the item Culinary Techniques 4*,* which assessed confidence in using the microwave for cooking, was retained despite showing a weak factor loading in the EFA, as well as limited contribution to internal consistency (as previously discussed in Internal Consistency Reliability Results section). Given its conceptual relevance for university students and the expert feedback suggesting the need for clearer wording, the item was retained provisionally after being reworded. Its performance should be further examined in future validation studies.

Following these adjustments, the EFA was repeated using the refined item pool. This process resulted in the development of the fourth version of the SCSEQ (S4 File).

### Test-retest reliability

The ICC for the total SCSEQ score was 0.91, 95% CI [0.80, 0.96], indicating excellent agreement between Time 1 and Time 2. ICC values for advanced cooking self-efficacy (Factor 1) and basic cooking self-efficacy (Factor 2) were 0.91 and 0.80, respectively. Pearson correlations also indicated strong and statistically significant associations between the two time points for both factors: advanced cooking self-efficacy (Factor 1), r = 0.91, 95% CI [0.80, 0.96], p < .001; and basic cooking self-efficacy (Factor 2), r = 0.81, 95% CI [0.60, 0.91], p < .001. The total SCESQ score also showed a strong correlation across the two time points, r = 0.91, p < .001. Together, the ICC estimates and supplementary Pearson correlations provide preliminary evidence of temporal stability over the two-week interval [49]. To examine whether voluntary dropout may have inflated these estimates, Time 1 scores and demographic characteristics were compared between the 28 retest completers with identifiable codes and the 45 non-completers. No statistically significant differences were found for the total SCSEQ score (completers: M = 3.72, SD = 0.60; non-completers: M = 3.64, SD = 0.38; t(71) = 0.75, p = .457), Factor 1 (t(71) = 0.72, p = .473), Factor 2 (t(71) = 0.63, p = .534), age (t(71) = −0.14, p = .890), gender distribution (χ²(2) = 0.31, p = .857), or living arrangement (χ²(2) = 1.52, p = .468). These results provide preliminary evidence that differential attrition did not systematically bias the temporal stability estimates.

## Discussion

This study presents the development and psychometric evaluation of the SCSEQ, the first theory-driven instrument specifically designed to assess cooking self-efficacy among Spanish university students. Despite the growing recognition of cooking self-efficacy as a modifiable factor for promoting healthier dietary behaviours [9,50,51–53], no existing instrument has been developed, translated, or cross-culturally adapted to evaluate the outcomes of culinary interventions grounded in SCT within this specific population.

Existing instruments present several limitations that constrain their applicability to this specific population and cultural setting. Many were developed for general adult populations or for university settings in culturally distinct contexts (e.g., Anglo-Saxon countries) [54]. Others were overly lengthy or designed exclusively for in-person delivery, limiting their adaptability to online or hybrid intervention formats. Additionally, some included domains beyond the core construct of cooking self-efficacy, such as nutrition knowledge or label reading. Several instruments also contained items requiring culinary skills that are unrealistic for many university students living with limited time, budgets, and shared kitchen resources. For example, items assessing confidence in preparing sauces from scratch represent advanced tasks that are unlikely to increase students’ likelihood of cooking more frequently or be addressed in culinary interventions targeting this population [12].

Given the limitations of existing instruments, the SCSEQ was not derived from a single source. Instead, it was developed through a selective process of integrating, translating, and adapting items from three validated instruments [12]. The goal was to create a concise instrument that was culturally tailored to Mediterranean food practices and reflected students’ everyday cooking realities [12]. It is also flexible in format, suitable for both online and in-person interventions.

The development and preliminary evaluation process of the SCSEQ was iterative, with items added, revised, or excluded based on both statistical performance and theoretical relevance. This approach mirrors validation processes adopted in other cooking self-efficacy instruments [9,32], and helped ensure that the final version retained both conceptual relevance and preliminary psychometric adequacy.

During the face validity testing phase, Spanish nutrition and culinary experts supported the clarity and relevance of the items, suggesting minor revisions that enhanced readability and reduced ambiguity [40]. Internal consistency analysis showed high scale-level reliability (ω = 0.9), which provides promising preliminary evidence of coherence among the retained items given the small sample size (N = 73). EFA further provided initial support for an interpretable factor structure aligned with the theoretical construct of cooking self-efficacy, while also identifying items requiring further evaluation in larger samples. EFA suggested a two-factor solution distinguishing between lower- and higher-complexity culinary tasks relevant to this population. This finding is consistent with Costa et al., (2023) [29] who also observed variance in students’ confidence based on task difficulty. Moreover, our results support the idea that cooking self-efficacy may vary across different types and levels of cooking activities. Previous studies have similarly noted that cooking self-efficacy varies according to task difficulty [9,29,35]. Students tend to report greater confidence in basic tasks (e.g., boiling) and lower confidence in more demanding skills (e.g., cooking legumes or fish), highlighting the importance of accounting for task granularity in the design of cooking self-efficacy scales [29].

Beyond task complexity, the iterative evaluation process also highlighted the importance of distinguishing between perceived capability and environmental constraints. For example, the item assessing the availability of kitchen utensils (General 5) was excluded from the final EFA model because, from a theoretical standpoint, access to utensils reflects a structural or material condition rather than the psychological construct of cooking self-efficacy. In contrast, the item assessing microwave use (Culinary Techniques 4) was provisionally retained despite its suboptimal psychometric performance. Microwave cooking represents an accessible and contextually relevant culinary practice for university students, particularly for those sharing kitchens, living in temporary housing, or facing limited time and resources. Retaining this item, while excluding items that primarily capture material constraints, helped preserve the content validity of the SCSEQ by distinguishing students’ perceived psychological capability to cook from external structural barriers that may influence cooking behaviour.

Finally, test–retest analyses provided preliminary evidence of temporal stability over the two-week interval. ICC estimates should be interpreted alongside Pearson correlations and with caution given the relatively small retest subsample. These findings are consistent with prior validation studies of cooking self-efficacy and culinary skills instruments [30] and suggest that the SCSEQ may be suitable for repeated use in intervention settings.

Overall, this study provides initial evidence of the reliability and validity of the SCSEQ in assessing cooking self-efficacy among Spanish university students. The use of this instrument will enable researchers and educators to evaluate the outcomes of culinary and nutrition interventions in this population and support the design of programs that promote healthier eating habits during this formative stage of life.

This study has several limitations that should be acknowledged. First, convergent and discriminant validity were not assessed due to time constraints and because comparator measures were not included in this pilot phase [40]. Future research should incorporate these psychometric evaluations along with known-groups validity to further strengthen the validation of the SCSEQ. Second, this study had a relatively small sample size (N = 73). While standard recommendations for exploratory factor analysis suggest a minimum of 150 participants or a ratio of 10–20 participants per item, smaller samples can be acceptable when factor loadings are strong and the scale demonstrates internal consistency [42]. A related limitation is the absence of confirmatory factor analysis. Because the two-factor structure was derived from EFA in the same pilot sample used for item refinement, it should not be interpreted as confirmed. Future studies should test this structure using CFA in an adequately powered independent sample. Third, participation in the study was voluntary, which may have introduced self-selection bias. Fourth, only 28 participants completed the retest assessment, which may limit the precision and generalizability of temporal stability estimates. Although our attrition analysis revealed no systematic selection bias between completers and non-completers, future studies should include larger retest samples to ensure greater statistical power. Future studies should include larger retest samples. Fifth, most participants lived with their families, which may limit the generalizability of the findings to students living independently or those fully responsible for food provisioning. Living arrangements may influence students’ exposure to cooking tasks, access to kitchen resources, meal planning responsibilities, and perceived cooking self-efficacy. Future validation studies should recruit more balanced samples by living arrangement and examine whether the SCSEQ performs similarly across students living with family, with peers, or alone. Finally, the SCSEQ was developed and validated in Spanish and tailored to the cultural and culinary context of Spain. Cross-cultural adaptation and validation in other linguistic and sociocultural settings will be necessary to assess the applicability and reliability of the questionnaire beyond the Spanish university context [30].

## Conclusions

This study developed and evaluated the SCSEQ, the first instrument specifically designed to assess cooking self-efficacy among Spanish university students. The SCSEQ was built around the unique needs, motivators, and barriers experienced by students in Spain. As a result, the instrument offers greater ecological validity for evaluating culinary interventions designed for this population. The final 25-item version of the SCSEQ showed promising preliminary psychometric properties, including high internal consistency, evidence of temporal stability, and an interpretable exploratory two-factor structure. This structure distinguished between basic and advanced cooking self-efficacy, suggesting that students’ perceived culinary competence may vary according to the complexity of cooking tasks. Designed in a concise and targeted format, the SCSEQ offers a practical and scalable instrument for researchers, educators, and public health practitioners to evaluate hybrid or digital SCT-based culinary and nutrition interventions. Further research is needed to confirm the factor structure through CFA in a larger independent sample and to assess additional forms of validity, including convergent, discriminant, and known-groups validity.

## Supporting information

S1 File Version 1 of the SCSEQ (for face-validity). (DOCX)

S2 File Version 2 of the SCSEQ (for pilot testing). (DOCX)

S3 File Version 3 of the SCSEQ (after internal consistency reliability analysis). (DOCX)

S4 File Final version of the SCSEQ (after exploratory factor analysis). (DOCX)

S5 File SCSEQ validation study. (XLSX)
