# Supplementary material for: Development and psychometric evaluation of the Spanish Cooking Self-Efficacy Questionnaire (SCSEQ) for Spanish University Students
Source: PLoS One. 2026 Jul 8;21(7):e0352758. doi: 10.1371/journal.pone.0352758 (PMC13345255; doi:10.1371/journal.pone.0352758)
Supplement: S1 File — (DOCX) [file pone.0352758.s001.docx]

**SUPPORTING INFORMATION S1**

**VERSION 1 OF THE SCSEQ (for face-validity)**

**For each item below, please indicate the extent to which you agree with these statements**

**(**Strongly disagree (1); Disagree (2); Neither agree nor disagree (3); Agree (4); Totally agree (5))

| **Coding** | **Item** |
| --- | --- |
| General_1 | I feel limited in the kitchen due to my lack of culinary knowledge. |
| General_2 | When I cook, I feel prepared to manage any unexpected event. |
| General_3 | When I cook, I feel like I can solve problems that arise with little effort. |
| General_4 | I know how to use the kitchen utensils and equipment I have. |
| General_5 | When I buy food, I already know that I am going to cook. |
| General_6 | I feel capable of cooking with the ingredients I have at home. |
| General_7 | Before I start cooking, I usually have a mental plan of what I want to make. |

| **Comment** | **Pertinence** | **Clarity** |
| --- | --- | --- |
|  |  |  |

**Please indicate how qualified you are to cook with the different food groups** (Very little confident (1); Poorly confident (2); Neither highly confident nor poorly confident (3); Confident (4); Very confident (5)):

| **Coding** | **Item** |
| --- | --- |
| Food groups_1 | Vegetables |
| Food groups _2 | Pasta |
| Food groups _3 | Rice |
| Food groups _4 | Tubers (potato, sweet potato or beet) |
| Food groups _5 | Legumes (including soy and derivatives) |
| Food groups _6 | White meats |
| Food groups _7 | Red meat |
| Food groups _8 | Fish |
| Food groups _9 | Eggs |

| **Comment** | **Pertinence** | **Clarity** |
| --- | --- | --- |
|  |  |  |

**Indicate how qualified you feel to perform these culinary techniques** (Very little confident (1); Poorly confident (2); Neither highly confident nor poorly confident (3); Confident (4); Very confident (5)):

| **Coding** | **Item** |
| --- | --- |
| Culinary techniques_1 | Stew (cook for long periods of time, at least an hour in a liquid or sauce). Example: beef in sauce. |
| Culinary techniques _2 | Boil (any type of food: rice, pasta, eggs, etc. ) |
| Culinary techniques _3 | Steam cooking (the food never touches the water, it is made with the steam itself) |
| Culinary techniques _4 | Steam cooking using the microwave |
| Culinary techniques _5 | Roasting food in the oven, for example, raw meat or fish, vegetables, etc. |
| Culinary techniques _6 | Fry in a pan or wok with oil. |
| Culinary techniques _7 | Pre-prepare raw vegetables (peel and cut an onion, a carrot, prepare a broccoli for cooking, etc. ). |
| Culinary techniques _8 | Pre-prepare meat (debone a chicken, make some burgers , make some meatballs, etc. ) |
| Culinary techniques _9 | Pre-prepare fish (remove the bones, clean it and prepare it for cooking). |
| Culinary techniques _10 | Dress or season food (use herbs or spices to flavor dishes). |

| **Comment** | **Pertinence** | **Clarity** |
| --- | --- | --- |
|  |  |  |

**For each item below, indicate the extent to which you agree with these statements in relation to kitchen resource management (**Strongly disagree (1); Disagree (2); Neither agree nor disagree (3); Agree (4); Totally agree (5)):

| **Coding** | **Item** |
| --- | --- |
| Culinary resources_1 | I feel able to double recipes to cook for more than one day. |
| Culinary resources _2 | I feel capable of preparing healthy dishes with few ingredients. |
| Culinary resources _3 | I feel capable of preparing healthy dishes with little time. |
| Culinary resources_4 | I feel able to use leftover food from other days to create a dish. |

| **Comment** | **Pertinence** | **Clarity** |
| --- | --- | --- |
|  |  |  |

**For each item below, indicate the extent to which you agree with these statements regarding organization and planning (**Strongly disagree (1); Disagree (2); Neither agree nor disagree (3); Agree (4); Totally agree (5)):

| **Coding** | **Item** |
| --- | --- |
| Planning and organizing _1 | I feel capable of cooking one day for the entire week. |
| Planning and organizing _2 | I feel able to properly plan the purchase based on what I want to cook. |

| **Comment** | **Pertinence** | **Clarity** |
| --- | --- | --- |
|  |  |  |

**GENERAL QUESTIONS ABOUT YOU**

**Please indicate if you are...**

▢ Man (1)

▢ Woman (2)

▢ Nonbinary (3)

▢ I prefer not to answer (4)

**Indicate the university degree you are pursuing**

____________________________

**Indicate your general state of health.**

▢ Bad (1)

▢ Regular (2)

▢ Good (3)

▢ Very good (4)

▢ Excellent (5)

**Please indicate your age in years:** ____

**What is your birthplace?**

Spain

Other country (specify): Specify year of arrival:

| **Comment** | **Pertinence** | **Clarity** |
| --- | --- | --- |
|  |  |  |
