# Supplementary material for: Development and psychometric evaluation of the Spanish Cooking Self-Efficacy Questionnaire (SCSEQ) for Spanish University Students
Source: PLoS One. 2026 Jul 8;21(7):e0352758. doi: 10.1371/journal.pone.0352758 (PMC13345255; doi:10.1371/journal.pone.0352758)
Supplement: S3 File — (DOCX) [file pone.0352758.s003.docx]

**SUPPORTING INFORMATION S3**

**VERSION 3 OF THE SCSEQ (after internal consistency reliability analysis)**

Deleted due to weak loads in the internal consistency analysis:

- When I buy food, I already know what I'm going to cook. (general 1)
- Fruit (food groups 2)
- Red meat (food groups 7)
- Resources 3 (I feel capable of preparing healthy dishes with little time.)

Rewritten: Microwave (marked in orange)

**For each item below, please indicate the extent to which you agree with these statements** (Strongly disagree (1); Disagree (2); Neither agree nor disagree (3); Agree (4); Totally agree (5)):

| **Coding** | **Item** |
| --- | --- |
| General_1 | When I buy food, I already know what I'm going to cook. |
| General_2 | I feel limited in the kitchen due to my lack of culinary knowledge. |
| General_3 | I feel capable of cooking with the ingredients I have at home. |
| General_4 | When I cook, I feel prepared to handle problems or incidents that arise, such as missing an ingredient from the recipe and having to improvise. |
| General_5 | In my kitchen, I have all the utensils I need to make any kind of recipe. |
| General_6 | I feel prepared to read or watch and try new recipes. |

**Please indicate how qualified you are to cook with the different food groups** (Very little confident (1); Poorly confident (2); Neither highly confident nor poorly confident (3); Confident (4); Very confident (5)):

| **Coding** | **Item** |
| --- | --- |
| Food groups_1 | Vegetables |
| Food groups _3 | Cereals (pasta, rice, quinoa, etc). |
| Food groups _4 | Tubers (potato, sweet potato or beet) |
| Food groups _5 | Legumes (including soy and derivatives) |
| Food groups _6 | White meats |
| Food groups _8 | Fish |
| Food groups _9 | Eggs |

**Indicate how qualified you feel to perform these culinary techniques** (Very little confident (1); Poorly confident (2); Neither highly confident nor poorly confident (3); Confident (4); Very confident (5)):

| **Coding** | **Item** |
| --- | --- |
| Culinary techniques_1 | Stew (cook for long periods of time, at least an hour in a liquid or sauce). Example: beef in sauce. |
| Culinary techniques _2 | Boil (any type of food: rice, pasta, eggs, etc. ) |
| Culinary techniques _3 | Steam cooking (the food never touches the water, it is made with the steam itself) |
| Culinary techniques _4 | Cook with the microwave (steam cooking, lekue, grill, etc) |
| Culinary techniques _5 | Roasting food in the oven (raw meat or fish, vegetables, etc.) |
| Culinary techniques _6 | Fry in a pan or wok with oil (potatoes, vegetables, breaded meat, etc). |
| Culinary techniques _7 | Grill (cook on a very hot surface with little oil). |
| Culinary techniques _8 | Pre-prepare raw vegetables (peel and cut an onion, a carrot, prepare a broccoli for cooking, etc. ). |
| Culinary techniques _9 | Pre-prepare meat (debone a chicken, make some burgers , make some meatballs, etc. ) |
| Culinary techniques _10 | Pre-prepare fish (remove the bones, clean it and prepare it for cooking). |
| Culinary techniques _11 | Dress or season food (use herbs or spices to flavor dishes, prepare vinaigrettes and/or sauces). |

**For each item below, indicate the extent to which you agree with these statements in relation to kitchen resource management** (Strongly disagree (1); Disagree (2); Neither agree nor disagree (3); Agree (4); Totally agree (5)):

| **Coding** | **Item** |
| --- | --- |
| Culinary resources_1 | I feel capable of adapting recipes to the amount of food I want to prepare (for more people or for more days). |
| Culinary resources _2 | I feel capable of preparing healthy dishes with few ingredients. |
| Culinary resources_4 | I feel able to use leftover food from other days to create a dish. |

**For each item below, indicate the extent to which you agree with these statements regarding organization and planning** (Strongly disagree (1); Disagree (2); Neither agree nor disagree (3); Agree (4); Totally agree (5)):

| **Coding** | **Item** |
| --- | --- |
| Planning and organizing _1 | I feel capable of cooking one day for the entire week. |
| Planning and organizing _2 | I feel able to properly plan the food shopping list based on what I want to cook. |
